# Turning metaphor on its head: a “target-to-source transformation” approach in statistics education

**DOI:** 10.3389/fpsyg.2023.1162925

**Published:** 2023-06-09

**Authors:** Dennis Tay

**Affiliations:** Department of English and Communication, The Hong Kong Polytechnic University, Kowloon, Hong Kong SAR, China

**Keywords:** metaphor, pedagogy, statistics education, regression analysis, target-to-source transformation

## Abstract

Many practical applications of metaphors are based on the idea that they are static TARGET IS SOURCE structures that support unidirectional meaning transfer for various purposes. Examples include healthcare and education where metaphors build cognitive and communicative bridges between the abstract and concrete. However, real-world metaphor use is often more dynamic than static, raising the question of how practical applications could benefit from a more correspondingly dynamic perspective. Drawing upon learning models that view learner output as creative transformations of input, this article introduces a “target-to-source transformation” approach that (i) initially frames concepts unfamiliar to novice learners as metaphorical targets as per received wisdom, but after some time, and (ii) invites learners to transform these targets into source domains for new target domains of their choosing. A pilot implementation is reported in the context of a statistics course, in particular the concept of regression analysis, for humanities students. Examples of transformed metaphors include different aspects of regression as sources for creative targets like “arranging a meeting time for friends,” “finding a life partner,” and “fortune-telling.” Analysis of these examples suggests that the approach creates a sense of pedagogical consistency, allows students to exercise creativity, and gives teachers novel insights into their level of understanding. Points for critical reflection will also be raised for future development of the approach, including the need to consider oft-overlooked metalinguistic attitudes held by laypersons toward metaphors.

## 1. Introduction

Three of the most prominent constructs in contemporary metaphor research are the source, target, and mappings between the two. The highly influential Conceptual Metaphor Theory (Lakoff, 1993), for example, defines “conceptual metaphors” as systematic unidirectional mappings from source to target domains. Expressed in the form TARGET IS SOURCE, conceptual metaphors have become fundamental units of analysis and provide a dominant theoretical frame for metaphor research and application. Examples include descriptions of conceptual metaphors in different languages (Yu, 1998; Kövecses, 2005) and non-linguistic modes (Forceville and Urios-Aparisi, 2009) and experimental studies of their psychological reality (Gibbs, 1994; Boroditsky, 2001; Glucksberg, 2003), as well as the functions and implications of source-to-target metaphorical inferencing in contexts such as politics (Musolff and Zinken, 2009), healthcare (Tay, 2013; Demjén et al., 2019), and marketing (Burgers et al., 2015). For instance, in mental healthcare contexts, clients' conceptualizations of their issues are typically framed as target domains that can be better communicated, understood, or even replaced with source domains that are deemed more “adaptive” (Kopp and Craw, 1998; Stott et al., 2010). Many education researchers and practitioners hold a similar view of metaphors as cognitive bridges that help learners connect a body of “source knowledge” that is more familiar, vivid, or concrete, with new “target” knowledge that is less so (Duit, 1991). Metaphors are deemed to be especially useful when learners have minimal knowledge (Donnelly and McDaniel, 2000), or when the target knowledge is itself still in its formative stages (Boyd, 1993; Holyoak and Thagard, 1995). This philosophy of distinctively framing and transferring inferences from sources to targets has been evident in both the sciences (Gentner and Gentner, 1983; Tabor-Morris et al., 2009) and humanities (Cameron, 2003; Littlemore, 2009).

The above treatments of metaphor as a static source–target structure have not gone unchallenged. For example, from observing the “complex dynamics of real-world language use in social situations” (Cameron et al., 2009; p. 64), Cameron and associates offer an alternative view of metaphor as a fluid process rather than a static structure. Real-world metaphor is better described as constantly shifting source and target fragments infused with semantic and pragmatic features that emerge from the context of use (Cameron and Deignan, 2006; Cameron and Maslen, 2010). Similar observations of the dynamic behaviors of metaphor have been made in contexts ranging from scientific to business and healthcare discourse, each of which may occur at different time scales. While fundamental conceptual metaphors used to frame important social issues like climate change may undergo subtle changes over a long period (Nerlich and Jaspal, 2012), the strategic interplay between sources and targets may occur over short interactional spans in contexts like psychotherapy (Tay, 2014b; Tay and Jordan, 2015). A specific example relevant to this article is Tay (2014b) analysis of metaphors used by earthquake victims to relate their traumatic experiences. Descriptions of bodily experiences (e.g., *the ground was still moving and we were in the dark*), which would have been treated as target domains as per the conventional psychotherapy technique described above, turn out to be creatively utilized as source domains for other issues as the interaction unfolded (e.g., *we were in the dark and we didn't know where the future was going*) because of their “embodied” nature as theorized in CMT (Lakoff and Johnson, 1999). What then emerges is an interesting “chaining” dynamic where topics are transformed from targets into sources. The notion of “topic-triggered metaphors” first observed in business discourse (Koller, 2004) is in the same vein, as certain source domains are pragmatically motivated by the topic at hand rather than some ostensible static conceptual metaphorical structure.

**Figure 1:**
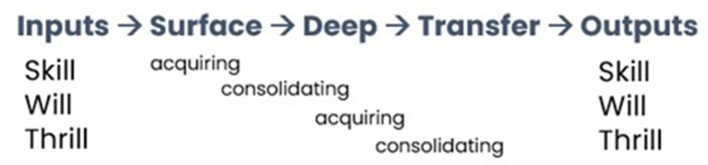
Three-phased model of learning.

The static vs. dynamic perspectives on metaphor raise the question of whether applications in fields such as psychological counseling and education, which have leaned mostly on the static perspective, could benefit by considering the dynamic. With the chaining of metaphor described above as a point-of-departure, this article describes an approach where concepts unfamiliar to novice learners, which are first introduced as target domains in typical pedagogical metaphors, are subsequently transformed by learners into source domains for a new target domain of their choosing. This learner-led process of (re)mapping a transformed source onto new targets, which we call “target-to-source transformation,” has the potential to serve as a creative exercise that furthermore provides insight into the extent of learners' conceptual mastery. As general theoretical motivation, consider the fact that contemporary theoretical models of learning tend to highlight some trajectory where input knowledge undergoes a gradual transformation process that leads to learners realizing its relevance for new situations. An example is Hattie and Donoghue (2016) three-phase model of learning, reproduced in a simplified form as Figure 1.

In this model, various inputs to learning are summarily related to the learner's skill (i.e., prior achievements), will (mental dispositions toward learning), and thrill (motivations held). They are also deemed as ideal outputs in that increased skill is “as valuable as enhancing the dispositions toward learning and … inviting students to reinvest more into their mastery of learning” (Hattie and Donoghue, 2016; p. 2). The learner then experiences “surface” and “deep learning,” both of which involve sub-phases of acquisition and consolidation. Surface learning refers to rote learning without conscious reflection on purpose or strategy, which may be consolidated by rehearsing the material to facilitate longer-term retention. On the other hand, deep learning is attested by seeking meaning, looking for patterns and principles, and relating and extending ideas across aspects of knowledge. The consolidation of deep learning occurs through critical self-questioning, monitoring, collaboration, problem-solving, and so on. Surface and deep learning may occur simultaneously for learners with strong metacognition and may also form a continuous cycle. Lastly, successful learning is marked by learners' willingness to transfer knowledge from one situation to another, which requires them to realize that the second situation resembles (or is perceived to resemble) the first situation. The conceptual connection between transfer and metaphor is clear, with metaphor being an obvious mechanism for transferring learned knowledge onto something new. Importantly, as we will see later, transfer does not require strong objective similarity or identity but can be motivated by more opportunistic perceptions of (di)similarities between the two things (Marton, 2006).

Relating this model more closely to metaphor, we can say that a typical pedagogical metaphor is well-chosen if (i) the source domain resonates with learners' existing knowledge and is able to engage their interest (i.e., skill, will, and thrill), (ii) complements the surface acquisition and consolidation of target knowledge, and (iii) facilitates deep learning by helping learners grasp the patterns, principles, and inferential logic underlying the target concept(s). The stage of the transfer, where these target concept(s) seek new grounds of application, is when the present proposal to initiate target-to-source transformation comes in. From the pedagogical perspective, this proposal aims to extend the ambit of metaphor as a teaching-and-learning tool where concepts are not just seen as static targets, but a creative source of conceptualization and reasoning especially at a stage where learners are expected to have developed some competence. Despite this creative expression, the approach can also foster a sense of pedagogical consistency when learners appreciate that the same tool (i.e., metaphor) used to impart the target concept(s) is redeployed for a different but related purpose of stretching their understanding. Several points of interest also arise from the perspective of metaphor theory, particularly the nature of metaphor chaining in an educational context. For example, it would be interesting to see how the original metaphor *given to* learners relates to the new metaphor *produced by* learners, which aspects are “carried over,” and how these are discursively constructed. This article reports a pilot implementation of our proposal in the context of basic statistics education, specifically the concept of regression analysis. The participants and method, including details of the original pedagogical metaphor and how the transformed metaphors are elicited, will be explained next. This is followed by analyzing three aspects of transformed metaphors and their theoretical and pedagogical implications: (ii) how transformed metaphors rely on their original sources, and (iii) misrepresentation as a diagnostic of students' conceptual understanding. Given the preliminary nature of the present findings, some future research directions are suggested in the concluding section.

## 2. Participants and method

This study took place in an undergraduate course (*N* = 50) in a language and communication degree program. The course introduced basic statistical knowledge for future careers in teaching, sales and marketing, and the media. All students were considered novice learners as they had no prior systematic training in statistics. After 2 weeks of basic descriptive statistics and an introduction to hypothesis testing, the next few sessions focused on the following concepts of linear regression analysis.

- Ordinary least-square regression applied to one predictor and one outcome variable.
- The total sum of squares (SST), regression sum of squares (SSR), and error sum of squares (SSE), and their relationship (SST = SSR + SSE).
- Manual calculation of SST, SSR, and SSE.
- The coefficient of determination R2 as a measure of model fit.

**Figure 2:**
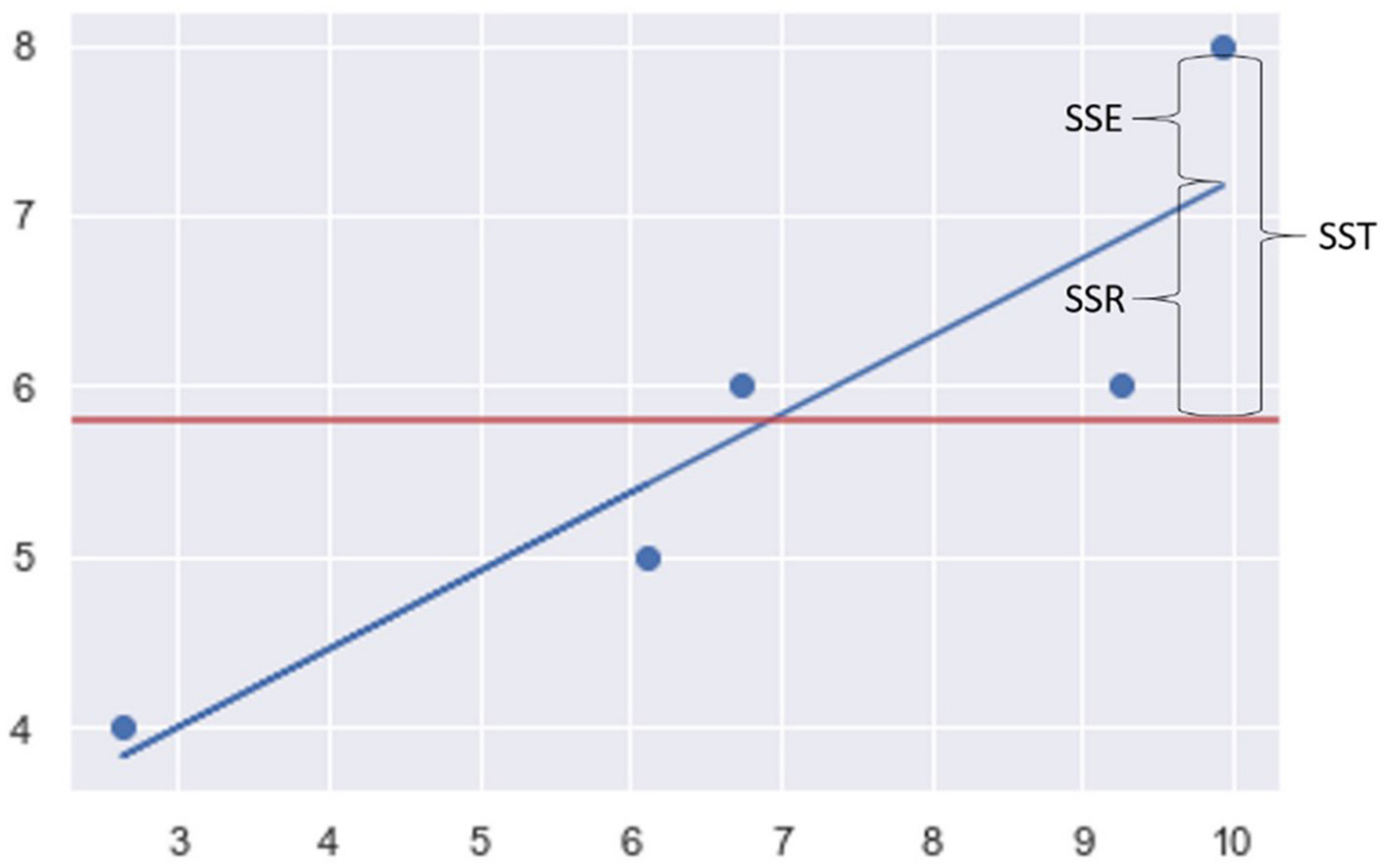
Standard regression instructional diagram.

Students were first exposed to the standard regression instructional diagram shown in Figure 2 to gain a general understanding of the above bullet points. As part of another study that compared the effect of different metaphors on assessment outcomes (Tay, 2022a), they were then randomly assigned and exposed to one of two pedagogical metaphors: REGRESSION ANALYSIS IS DOTS AND LINES IN PHYSICAL SPACE, or REGRESSION ANALYSIS IS A RADIO STATION BROADCASTING A SIGNAL (Martin, 2003). These metaphors are conveyed to the students in the following form.

### 2.1. Regression analysis is dots and lines in physical space

It is helpful to visualize *regression analysis* in terms of dots and lines in physical space. Each dot on the scatterplot represents a *student*. The distance between the dots, therefore, represents *how different their score and study hours are*. The further the distance between dots, the greater the *variability among students*. The horizontal line is the mean line. It represents the *mean exam scores* and divides the territory between the *above and below average students*. The total distance between the dots and the line is the *total variability in exam scores*. If the dots are close to the line, there is *less variability*, and the scores are easier to predict. However, if the dots are scattered far apart, there is *more variability*, and the scores are harder to predict. The straight regression line represents our regression *model*. Each point on the line represents the *predicted performance score* based on the number of study hours. The vertical distance between each dot and its corresponding point on the line represents the *error or residual*. The smaller the distance between the line and the dots, the *more accurate the prediction* is. From another perspective, we can also say that the total distance between points on the regression line and the mean line represents the *variability explained by the model*. In general, we want to minimize prediction errors and maximize the explained variability. In real life, it is almost impossible to have a model with absolutely no error, because there is always some unknown variability that cannot be explained.

### 2.2. Regression analysis is a radio station broadcasting a signal

It is helpful to visualize regression analysis in terms of a radio station broadcasting a signal. Imagine that I am listening to this broadcast from a long distance away. What I hear will not be identical to the original signal, because of some form of interference or signal degradation which we call “noise” in general. In other words, the signal I hear is actually made up of the original signal from the station plus this noise. Engineers will use different techniques and tools to make the signal I hear as close as possible to the original signal, or to reduce the noise. However, it is very difficult to achieve zero noise. Regression analysis follows the same principle. We are given data on the exam performance and number of study hours for a sample of students, and we want to see if there is a relationship between them, so that we can predict exam performance based on study hours for students not in the sample. This prediction will not be perfectly accurate because there is also “noise” in our data. The number of study hours may partly explain differences in performance, but there are other factors that we did not measure or expect. Just as engineers use different techniques and tools to reduce noise in the radio signal, regression analysts use different techniques and tools to optimize the predictions using the data available. Moreover, just as achieving zero signal loss is very difficult, in most situations it is almost impossible for predictions to have absolutely no error. This is because there is always some unknown variability that cannot be explained.

In addition to the source domains, the key difference between the two metaphors lies in their discursive construction. In the physical space metaphor, source elements (i.e., spatial descriptions) are emboldened and target elements (i.e., aspects of regression) are italicized to show their systematic juxtaposition, highlighting point-to-point correspondences rather than some overarching similarity between source and target (Gentner, 1983; Duit, 1991). This has been described as a “correspondence model” (Wee, 2005; Tay, 2022a). The radio station metaphor works the opposite way by not emphasizing point-by-point correspondences, but highlighting the overarching objective of both source and target (Glucksberg and McGlone, 1999) as reducing “noise” (emboldened). This has been described as a “class inclusion model” where the so-called overarching objective subsumes both source and target (Wee, 2005; Tay, 2022a). The effects of discursive construction on subsequent performance measures like calculation exercises and short conceptual essays were reported in Tay (2022a). For the present study, however, the two variant constructions simply served as different starting points for the next phase where students' transformed metaphors were elicited.

The critical phase of eliciting students' transformed metaphors took place at the end of the sessions on regression analysis. The instructor invited volunteers (*N* = 7) to an informal session to share their experiences of the course up to that point. Care was taken to ensure that both original pedagogical metaphors were “represented,” in that there would be at least one student exposed to either metaphor taking part in the sharing session. It was explained that the objective of the session was to collect feedback and that students' responses will be used for pedagogical research. After a preliminary discussion of their experiences, the prompt below was used to elicit their transformed metaphors. For students exposed to the radio station metaphor, “dots and lines in physical space” was replaced by “a radio station broadcasting a signal.” Students' responses were transcribed and minimally edited for grammatical errors.

We saw in the lectures that regression analysis can be compared to (dots and lines in physical space/a radio station broadcasting a signal). So, we used (dots and lines in physical space/a radio station broadcasting a signal) as a tool to understand regression better. Now that you have a basic understanding of regression, shall we try to flip things around and use regression as a tool to understand something else? What is this “something else” that regression can be compared to?

There are two important points to be made about the prompt. The first is the deliberate avoidance of technical terms like “metaphor” or “analogy,” and the second is the attempt to carefully deconstruct what is meant by metaphor. We can see that the prompt was designed to remind students about the nature, function, and form of the pedagogical metaphors they previously experienced. Their nature was to setup systematic comparisons between target and source to perform the function of enhancing conceptual understanding, and their formal structure X IS Y can be “flipped around” to produce Z IS X, where X is the original target, Y is the original source, and Z is the new target. Researchers in applied contexts like education (Wan, 2011) and psychotherapy (Tay, 2022b) have highlighted the important question of how to best elicit, or draw attention to, the presence and intended use of metaphors. This is especially important for layperson audiences who are unlikely to share the researcher's understanding despite the ostensible pervasiveness of metaphors, and who might resist or even display negative attitudes toward them (Tay, 2020). The present study, therefore, opts for a more careful approach that does not assume prior understanding of metaphor or attitudes toward it.

## 3. Results and discussion: three illustrative target-to-source transformations

The seven responses predictably varied in quality and content. Two students demonstrated difficulties with understanding the prompt, one appeared to question the usefulness of the activity, while the other provided semi-coherent answers requiring extensive follow-up prompts. Three other students offered thoughtful responses that will be analyzed below to illustrate the pedagogical plausibility of eliciting target-to-source transformations. Importantly, the varying quality of responses that arose from even a limited sample suggests the need for future work to consider how metalinguistic awareness and attitudes toward metaphor may impact pedagogical effectiveness. Consider, for example, the following (extracts of) less-than-ideal responses.

Sorry I don't totally understand what you mean by using regression as a tool. I thought we use regression to predict values for data, so what do you mean by using it to compare with something else?

Why do we need to talk about or understand something else? I think the course is about statistics and regression.

I think regression is a tool to understand data, like how an outcome is related to some variables. So the “something else” can be any social data, maybe not just the radio station example you said.

The first response illustrates what could happen when the desire to not assume a prior understanding of “metaphor” is met by a seeming case of inadequate metalinguistic awareness of it. The student has a correct understanding of the purpose of regression analysis, which demonstrates the effectiveness of originally framing it as a target domain. However, they appear unwilling and/or unable to perceive the underlying mechanism (of metaphor) that was used to enable this understanding, much less participate in further extensions of this mechanism. The second response is more explicitly resistive and questions the need to “talk about or understand something else,” suggesting a common pragmatic attitude toward learning where it is “enough” to just understand the target topic. The third response may appear semi-coherent from an idealistic metaphor-theoretical point of view, as the student seems to not understand “something else” as a counterpart conceptual structure in the same tidy way as many metaphor researchers do.

As mentioned above, such less-than-ideal responses highlight limitations to metaphor-related pedagogies and provide important food for thought we will revisit in the conclusion. For now, we return to the productive responses below and analyze them for their implications for metaphor theory and (statistics) pedagogy.

Example 1: “Arranging a time for friends to meet”

What I can think of is that when I am trying to arrange a time for my friends to meet, that is something like regression. You know that diagram with the dots and lines you showed us, I think maybe different people can be like all the dots or data points, who are all at different places and far away from each other, and when I arrange a meeting time I am trying to draw a line to connect everyone or make it as close as possible to everyone. Maybe the nearer the friends are to the line, that means those people will not be late for the meeting and there is less error like in regression. And those people on the line are those who can meet at the correct time. I think it's something like that, am I correct?

The student in Example 1 was exposed to the REGRESSION ANALYSIS IS DOTS AND LINES IN PHYSICAL SPACE metaphor, which they explicitly recall early on (“that diagram with the dots and lines…”). The target of the transformed metaphor is “arranging a time for friends to meet,” the main point being that finding the best time for an appointment between friends is like fitting a regression line of best fit for a set of data points. Each friend is likened to a data point occupying different spatial locations. The notion of error or residuals is also recruited to represent the extent of lateness for the appointment. Fairly characteristic of novice learners, they express some uncertainty about the “correctness” of the response at the end.

Example 2: “Finding a life partner”

I think the regression analysis reminds me of someone who wants to find a life partner, like a husband or wife. I don't know, so maybe marriage is something like a regression for me. I think the regression model you talked about is maybe like a set of criteria that you have for a husband. The different people you meet in your life are like the data points and they are all different because like the data points with different values they have different qualities or characters. Then maybe using the model or my criteria I can try to predict whether a new person I meet can fulfill the criteria I have? And if he is suitable that means he will be on the line, and if not suitable then there will be like an error. But my idea is not very clear I think.

The student in Example 2 was likewise exposed to the REGRESSION ANALYSIS IS DOTS AND LINES IN PHYSICAL SPACE metaphor, though this was not explicitly referenced. The target of the transformed metaphor is “finding a life partner” where different potential partners are likened to data points and the “set of criteria” for a life partner is likened to the regression line of best fit. The notion of error was likewise used to represent the extent of potential partners' suitability, just like in Example 1. Additionally, missing from Example 1 but present here, the important notion of predicting outcomes for future data was also recruited to express the idea of judging if “a new person” would be suitable. Throughout the response, the student likewise expressed uncertainty, perhaps even more so than in Example 1.

Example 3: “Fortune-telling”

We learnt that the most important use for regression is prediction, and making the predictions as accurate as possible like that radio signal example you gave. I think another thing that involves prediction and is maybe also a bit like regression is like fortune-telling, fortune-tellers that try to predict your future. Usually, they will also ask for your data and personal information like date of birth, occupation, and so on, and then try to tell you a story about your future. But still, I think the two things are actually very different. I don't think the personal information can be considered as data for regression because they are all taken from the same person and on different aspects, but for regression each data point is from different people and on the same aspect. Also of course fortune-telling is not scientific and is more like guessing, but regression is a very systematic thing and is about finding patterns in a lot of data.

The student in Example 3 was exposed to the REGRESSION ANALYSIS IS A RADIO STATION BROADCASTING A SIGNAL metaphor, as explicitly referenced early on (“like that radio signal example”). The target of the transformed metaphor is “fortune-telling,” as the response focuses on explaining how the overarching feature of prediction applies to both activities. A salient difference between Example 3 and the previous two examples is the attention to the dissimilarities between fortune-telling and regression, with the explicit disclaimer that “the two things are actually very different.” In other words, the transformed metaphor is sanctioned by the overarching relevance of “prediction,” but subsequently self-challenged by specifying a series of mismatches between target and source.

### 3.1. Discursive construction of transformed metaphors

We now make some collective observations from the three transformed metaphors and discuss what they imply for metaphor theory as well as (statistics) pedagogy. Referring back to the three-phase learning model in Figure 1, these transformed metaphors can be seen as preliminary evidence of student learning outputs, to be further developed as part of more complete teaching and learning activities designed in the future. We begin with what is apparent from the surface—the discursive elements that construct the transformed metaphors, which may provide clues on how students understand and convey important source–target relationships (cf. Tay, 2010). The first observation is that the structure of the students' metaphors appears to mirror what they were initially exposed to—described above as either a correspondence (REGRESSION ANALYSIS IS DOTS AND LINES IN PHYSICAL SPACE) or class inclusion structure (REGRESSION ANALYSIS IS A RADIO STATION BROADCASTING A SIGNAL). In correspondence structures, source and target elements are systematically juxtaposed to highlight point-to-point correspondences, ostensibly in order to guide recipients to make these important connections. We catch glimpses of this systematicity in Examples 1 and 2 despite their spontaneous nature. For example, consider the following short snippets from the original pedagogical metaphor, Example 1 and Example 2, respectively. Like in the original metaphor, source elements are emboldened and target elements are italicized to show this systematicity.

### 3.2. Original metaphor

It is helpful to visualize *regression analysis* in terms of dots and lines in physical space. Each dot on the scatterplot represents a *student*. The distance between the dots, therefore, represents *how different their score and study hours are*. The further the distance between dots, the greater the *variability among students*…

Example 1

You know that diagram with the dots and lines you showed us, I think maybe *different people* can be like all the dots or data points, who are all *at different places and far away from each other*, and when I *arrange a meeting time* I am trying to draw a line to connect everyone or make it as close as possible to everyone…

Example 2

I think the regression model you talked about is maybe *like a set of criteria that you have for a husband*. The *different people you meet in your life* are like the data points and they are all different because like the data points with different values they *have different qualities or characters.*..

It is evident from both Examples 1 and 2 that the general expository-style structure of intermittent source element–target element (or vice versa) pairs seem to have been preserved from the original metaphor. The same can be observed for the class inclusion structure experienced by the student in Example 3, as seen from the snippets below. Recall that the class inclusion structure does not emphasize point-by-point correspondences, but summarily highlights an overarching point that subsumes both source and target. This overarching point is emboldened in the snippets.

Original metaphor

... The number of study hours may partly explain differences in performance, but there are other factors that we did not measure or expect. Just as engineers use different techniques and tools to reduce noise in the radio signal, regression analysts use different techniques and tools to optimize the predictions using the data available.

Example 3

We learnt that the most important use for regression is prediction, and making the predictions as accurate as possible like that radio signal example you gave. I think another thing that involves prediction and is also a bit like regression is like fortune-telling, fortune-tellers that try to predict your future…

Similarly, just as the original metaphor delivers the overarching point after some (not necessarily intermittent) explanation of the source and target, the student in Example 3 emphasizes the common feature of “prediction” in both regression and fortune-telling in lieu of explicating systematic correspondences between the two. This initial observation of structural consistency between what was received (i.e., the original metaphor) and created (i.e., the transformed metaphor) may suggest that, despite the creative exercise of transferring knowledge to new situations via metaphor, the present students still seem to be guided (or constrained, depending on one's pedagogical perspective) by an implicit template that shaped the original teaching material. Recall that these “templates,” or correspondence vs. class inclusion structures, were designed to be maximally contrastive for experimental purposes. It is an open question whether such discursive behaviors are observable in other learner populations, and if so, to what extent they are helpful.

The second observation on discursive construction is the salient expression of uncertainty across all three examples. This includes typical hedging expressions like *maybe, I think, I don't know*, as well as more explicit disclaimers like *am I correct?* (Example 1) and *but my idea is not very clear* (Example 2). It is of course unsurprising for novice learners to express doubt about the “correctness” of their answers, which in this case may relate to their understanding of regression and/or self-perceived quality of their transformed metaphors. However, at a more subtle level, the expression of uncertainty could involve questioning the validity/purpose of using the metaphor itself. Similar to extended metaphors used by therapists and clients in psychotherapy (Tay, 2011), the preliminary samples indicate that hedging expressions tend to preface the introduction of source/target elements or statements of cross-domain mappings. This is apparent in the following extracts from Examples 1 and 2 (hedging expressions are emboldened).

Example 1

You know that diagram with the dots and lines you showed us, I think maybe different people can be like all the dots or data points… Maybe the nearer the friends are to the line, that means those people will not be late…

Example 2

I think the regression analysis reminds me of someone who wants to find a life partner, like a husband or wife. I don't know, so maybe marriage is something like a regression for me…The different people you meet in your life are like the data points and they are all different because like the data points with different values they have different qualities or characters. Then maybe using the model or my criteria I can try to predict whether a new person…

When produced by institutional “advice-givers” like therapists and teachers, such hedges may be intended to communicate the inherently approximate nature of metaphors, and the importance of distancing them from relevant literal facts (Tay, 2014a). However, when produced by counterparts like learners at key locations like sources/targets/mappings, hedges are equally likely to express doubts about whether such metaphors are “correct” as ostensible representations of technical concepts. This is again related to the aforementioned importance of not making naïve assumptions about layperson attitudes to metaphors, across the many contexts of applied metaphor research.

### 3.3. Metaphor chaining: reliance on the original source

**Figure 3:**
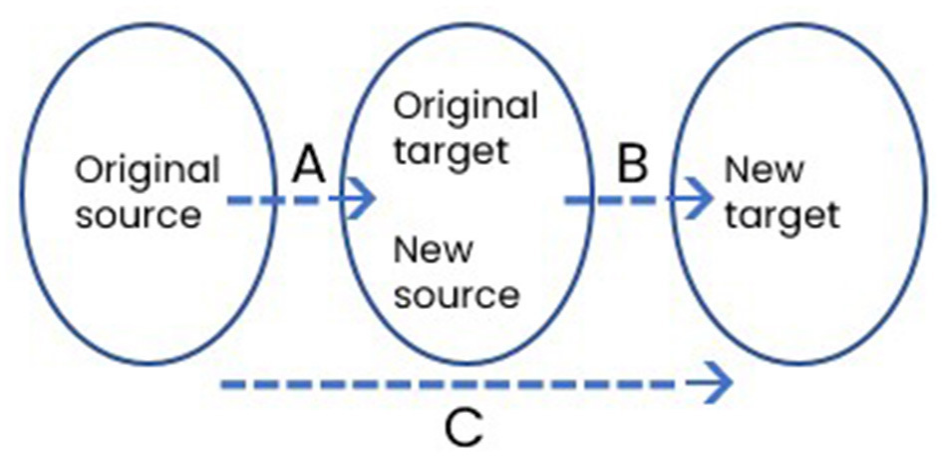
Schematic representation of a metaphor chain.

Moving from discursive construction to conceptual representation, the next observation relates to the nature of what we called metaphor chaining, where original topics turn from targets into sources for new topics. There are perceptible differences between the present examples and metaphor chains in the previously mentioned descriptions of earthquake experiences, as schematically captured in Figure 3.

In earthquake descriptions like *we were in the dark, we didn't know where the future was going*, the original target of being (literally) in the dark could have been described with a source but did not rely strongly on one as it was already “embodied” in the cognitive linguistic sense (Tay, 2014b). This corresponds to a weak mapping set A in Figure 3. However, mapping set B is strong as the speaker turns “being in the dark” into a new source for the new target of uncertainty about the future. There is also no obvious direct conceptual link between the original source and the new target, meaning mapping set C is also weak.

In present examples, however, mapping sets A to C all appear to be strong, resulting in a more integrated metaphor-chaining dynamic. In Examples 1 and 3, the students explicitly recall original sources—*you know that diagram with the dots and lines you showed us*, and *like that radio signal example you gave*—when presenting their new targets. More importantly, the inferential structure defining A seems to have been strongly preserved in B, which is equivalent to a strengthened C. In Examples 1 and 2 below, the dots and lines in the original source that represented analytical units in the regression were directly invoked to construe the new targets.

… that diagram with the dots and lines you showed us, I think maybe different people can be like all the dots or data points…

… different people you meet in your life are like the data points and they are all different because like the data points with different values…

As for Example 3, partly due to its class inclusion structure discussed above, all three domains—the original radio station source, regression, and the new fortune-telling target—were closely juxtaposed and linked to the overarching feature of ‘prediction'. Although this appeal to superordinate attributes has the effect of blurring the distinction between sources and targets (Wee, 2005), the sense of metaphor chaining or mutual reference between past taught contents and students' present creation is still clear.

…the most important use for regression is prediction, and making the predictions as accurate as possible like that radio signal example you gave. I think another thing that involves prediction and is also maybe a bit like regression is like fortune-telling…

The strong degree of reliance shown above echoes a point made in the previous section—that while students are able to creatively invent new targets that are *substantively* different, these targets are still *structurally* dependent on original sources, which is likely to create an impression of consistency throughout the learning phases. From a theoretical perspective, these initial observations lend support to the view that structural similarity might be a more important driver than substantive similarity in analogy creation and perception (Blanchette and Dunbar, 2000; Tay, 2021). Pedagogically, the spontaneous balance between creativity and pedagogical consistency can be interpreted as a point in favor of the present approach.

### 3.4. Misrepresentation as a diagnostic of conceptual understanding

The first two observations above are more descriptive, focusing on how students construe and communicate their transformed metaphors. Our final observation is more critical and pedagogically oriented in that we consider how these metaphors reflect students' (in)correct understanding of regression analysis. In other words, the misrepresentation of concepts via metaphor is a diagnostic of conceptual understanding. An examination of the described relationship between regression and each new target reveals various levels of understanding from Examples 1 to 3.

In Example 1, the metaphor of “arranging a time for friends to meet” reflected the student's recall and understanding of basic conceptual points about regression analysis. These include data points as mutually independent (*different people can be like all the dots or data points…*), occupying different positions (*who are all at different places…*), and the general idea of trying to minimize residuals when fitting a regression line (*the nearer the friends are to the line, that means those people will not be late for the meeting and there is less error…*). However, several critical elements appear to be misconstrued. The first is that there is no target domain counterpart of the predictors, or variables, that defined the position of the dots/friends in the first place. Second, the target domain also fails to capture the main idea of predicting future values, which is the purpose of the regression line. Instead, the regression line in the student's metaphor seems to be stipulating one singular time point for friends to meet, which is an incorrect analog of a different predicted value for each data point. It seems that the student has either incorrectly understood the above concepts or has chosen to paint a “looser” picture, aiming more at producing a coherent metaphor than a conceptually perfect one.

In Example 2, the metaphor of “finding a life partner” was likewise able to reflect the same basic conceptual points using a very different target (e.g., *the different people you meet in your life are like the data points… if not suitable then there will be like an error*). However, Example 2 may reflect a higher level of understanding because, at first, the concept of variables that defines the spatial location of the dots/persons was captured in the target domain (*like the data points with different values they have different qualities or characters*). Second, the concept of prediction was also captured by the notion of judging whether a new person (i.e., a new data point) falls on the line. Notwithstanding, just like in Example 1, it is apparent from the metaphor that some concepts have been misconstrued. Construing the regression model as a *set of criteria* that people should “strive toward,” rather than a mere description of how data points are connected, is technically incorrect. This may reflect the common misunderstanding that a statistical “model” represents an ideal rather than an empirical approximation of reality—something also seen in Example 1. Another subtle error is the construal of predictions (i.e., whether someone is suitable) as a yes/no categorical outcome rather than continuous values. Just like in Example 1, we have to consider the possibility that students are more focused on producing a coherent rather than a “correct” transformed metaphor.

In this regard, Example 3 appears to demonstrate the most mature understanding of regression analysis. Disclaimer statements like *I think the two things are actually very different* and *fortune-telling is not scientific and is more like guessing, but regression is a very systematic thing* are particularly telling. Not only does their elaboration reveal the sound understanding of specific technical points about regression (e.g., data taken from the same person vs. data taken from different people), but the act of disclaiming also suggests an awareness of the need to compromise between a coherent and technically correct metaphor.

## 4. Conclusion

This article introduced a teaching-and-learning approach that goes beyond representing new target concepts with metaphors to encourage novice learners to creatively transform these targets into sources after acquiring some basic competence. The approach was inspired by perceived gaps in applying a more dynamic perspective on metaphor to pedagogy, in accordance with learning models that advocate the student-led transfer of knowledge to new situations. Findings from a pilot implementation of this “target-to-source transformation” exercise in an undergraduate statistics course were reported. Examples of students' transformed metaphors were discussed in three aspects, ranging from their surface discourse construction to the construed conceptual relationships between the original source, the concept of regression analysis, and the new target.

The preliminary examples are neither representative of the present population of learners, nor generalizable to other populations. Their main value is to illustrate how the “target-to-source transformation” exercise could give students an opportunity to exercise creativity on a subject matter not normally associated with it. At the same time, a sense of pedagogical consistency was evident in student responses through the perceptible influence of the original metaphors on the transformed ones. The exercise also has the potential to give teachers novel insight into the extent of students' conceptual understanding. Nevertheless, these examples also raise some issues of concern. The fact that some students seem unable and/or unwilling to provide a coherent transformed metaphor, coupled with expressions of doubt about the validity of some responses, echoes the point observed elsewhere that laypersons' abilities, awareness, and attitudes about metaphor need to be critically considered before we prematurely assume that they “work.”

Given the constructive nature of the available responses, it is worth further developing and investigating the present proposal in several ways. The most obvious next step is to move from the research setting of sharing sessions back into the classroom and design the exercise more concretely, ideally as part of a class activity for individuals or groups. This would allow for a more systematic assessment of the effectiveness of “target-to-source transformation” for learning, for example by comparing understandings of regression between intervention and control groups. It should also provide more critical examples and insights into when, how, and why the approach does not work, how this might relate to attitudes and understanding toward metaphor, and contributing learner characteristics and factors. More optimistically, if transformed metaphors indeed reveal the extent of students' understanding, ways to factor them into assessment plans should be considered. The transformed metaphors could take on different creative forms, including visual or multimodal creations that would at the same time provide rich data for further research on the nature of metaphor chaining.

## Data availability statement

The original contributions presented in the study are included in the article/supplementary material, further inquiries can be directed to the corresponding author.

## Ethics statement

The studies involving human participants were reviewed and approved by the Hong Kong Polytechnic University. The patients/participants provided their written informed consent to participate in this study.

## Author contributions

The author confirms being the sole contributor of this work and has approved it for publication.
